# Predicting Risk-Taking Behavior from Prefrontal Resting-State Activity and Personality

**DOI:** 10.1371/journal.pone.0076861

**Published:** 2013-10-07

**Authors:** Bettina Studer, Andreas Pedroni, Jörg Rieskamp

**Affiliations:** 1 Department of Psychology, University of Basel, Basel, Switzerland; 2 Institute of Cognitive Neuroscience, University College London, London, United Kingdom; University of Medicine & Dentistry of NJ - New Jersey Medical School, United States of America

## Abstract

Risk-taking is subject to considerable individual differences. In the current study, we tested whether resting-state activity in the prefrontal cortex and trait sensitivity to reward and punishment can help predict risk-taking behavior. Prefrontal activity at rest was assessed in seventy healthy volunteers using electroencephalography, and compared to their choice behavior on an economic risk-taking task. The Behavioral Inhibition System/Behavioral Activation System scale was used to measure participants’ trait sensitivity to reward and punishment. Our results confirmed both prefrontal resting-state activity and personality traits as sources of individual differences in risk-taking behavior. Right-left asymmetry in prefrontal activity and scores on the Behavioral Inhibition System scale, reflecting trait sensitivity to punishment, were correlated with the level of risk-taking on the task. We further discovered that scores on the Behavioral Inhibition System scale modulated the relationship between asymmetry in prefrontal resting-state activity and risk-taking. The results of this study demonstrate that heterogeneity in risk-taking behavior can be traced back to differences in the basic physiology of decision-makers’ brains, and suggest that baseline prefrontal activity and personality traits might interplay in guiding risk-taking behavior.

## Introduction

If the same decision options with uncertain outcomes are presented to different people, they will rarely all make the same choice. Human risk-taking behavior varies considerably across individuals, both in the laboratory and in everyday life [1-5]. Such individual differences in risk-taking behavior might arise as a consequence of heterogeneity in basic brain physiology. The basic functional properties of an individual’s brain can be assessed by analyzing cortical activity at rest. This can for instance be accomplished using electroencephalography (EEG): Spectral power profiles in resting-state EEG recordings have high intraindividual stability and are reliable indicators of dispositional properties of brain function [6,7]. Two previous EEG-studies [8,9] assessed whether decision-making behavior is linked to cortical activity at rest, and provided somewhat divergent results. Schutter and Van Honk [8] measured participants’ resting-state activity before administering the Iowa Gambling Task (IGT) [10]. Overall task performance and the level of risk-taking were negatively correlated with theta-band power in electrodes overlying the frontal cortex. This result suggests that high power of theta oscillations in the bilateral (pre-)frontal cortex might disposition an individual to take frequent risks. Gianotti and colleagues [9] focused on interindividual variability in hemispherical balance in prefrontal resting-state activity, and found that stronger right-left asymmetry in prefrontal slow-wave power (theta and delta band) was associated with increased risk-taking on the Devil’s Task [11]. Thus, both previous studies demonstrate that individual differences in risk-taking behavior can be linked to resting-state slow-wave activity in the prefrontal cortex (PFC). However, the two studies yielded contradicting findings regarding the nature of this relationship between risk-taking and resting-state PFC activity. The data by Schuetter and Van Honk [8] suggests that risky choice behavior is linked to the resting-state activity in the *bilateral* PFC, while Gianotti et al. [9] observed a link with the *hemispherical balance* in the PFC. The first aim of the current study was to directly compare the predictive value of a) overall bilateral PFC activity at rest and b) the hemispherical balance in PFC resting-state activity for risk-taking behavior. We recorded resting-state brain activity in 70 healthy student volunteers using EEG, and analyzed slow-wave power (theta and delta band) in the prefrontal cortex. Risk-taking behavior was assessed subsequently to the resting-state recording with an economic decision making task.

Heterogeneity in the stable characteristics of individuals can also be described in a personality framework. Two personality characteristics that have been linked to decision-making behavior under uncertainty are sensitivity to reward and sensitivity to punishment. These traits are often quantified with the Behavioral Inhibition System/ Behavioral Approach System (BIS/BAS) scale [12]. The BIS/BAS scale is a self-report questionnaire designed to assess sensitivity of two neurophysiological motivation systems, which underlie human affect and behavior according to Gray’s theory of personality [13,14]: The BIS, which is sensitive to punishment and inhibits behavior that may have negative or painful consequences, and the BAS, which is sensitive to reward and promotes approach behavior. Recent studies found that scores on the BIS/BAS scale are predictive of individual’s performance on the IGT [15-18] and sensitivity to outcome probabilities [19]. In risky choice situations, high sensitivity to punishment should promote avoidance of potential losses and thereby lead to risk-averse behavior. In contrast, sensitivity to reward might lead to risk-seeking behavior, particularly if the risky option offers a potential for high gains. Thus, we predicted that risk taking on our task will be negatively correlated with BIS scores and positively correlated with BAS scores across participants.

While previous data indicate that both prefrontal resting-state activity and reinforcement sensitivity might explain some heterogeneity in risk-taking behavior, the relationship between these two predictors has rarely been explored. A number of previous studies reported a correlation between scores on the BIS/BAS scale and resting-state activity measured in frontal EEG electrodes [20-24]. These results suggest that BIS/BAS scores and EEG markers of prefrontal resting-state activity might reflect the same dispositional physiological properties of a decision maker. Meanwhile, recent data by Massar et al. [25] indicates that reinforcement sensitivity and prefrontal activity at rest might interplay to determine risk-taking behavior. In that study, the link between resting-state activity of the fronto-central electrodes and risk-taking behavior was modulated by scores on the BIS scale. For people with high BIS scores, theta-band power at rest significantly predicted risk-taking behavior on a subsequent gambling task, while no such relationship was found in people with low BIS scores. The second aim of the current study was to investigate the triangle between personality, prefrontal activity at rest and risk-taking behavior. More specifically, we aimed to test whether reinforcement sensitivity and PFC resting-state activity are directly related or interact in influencing risk-taking behavior.

## Materials and Methods

### Ethics statement

All participants provided written informed consent and this research was conducted in line with the Declaration of Helsinki. The study was approved by the Ethics Committee of Basel (protocol number 79/12).

### Participants

Seventy right-handed students (49 females; mean age = 24 years, SD = 3 years) took part in a single testing session, lasting approximately two hours. Participants had no history of neurological or psychiatric disorders and were pre-screened for drug and alcohol consume. Participants received course credits or 40 CHF for their participation, plus a bonus payment between 0 and 9 CHF depending on their earnings in the gambling task.

### Procedure

The testing session started with the preparation of the EEG, followed by a 5 minute long baseline EEG recording with alternating eyes-closed and eyes-open periods (40 and 20 seconds each, respectively). Next, participants completed a computerized gambling task assessing risk-taking behavior. EEG was recorded throughout the task (data not reported here). Participants were also administered the BIS/BAS scale [12] to measure trait reward and punishment sensitivity.

### EEG recording

EEG was acquired from 64 Biosemi active electrodes (Biosemi Instruments, Amsterdam, The Netherlands) positioned according to the standard 10-10 system montage. Electrooculogram (EOG) electrodes were placed on the outer canthi of the eyes and above and below the right eye. EEG and EOG were recorded at a sampling rate of 512Hz (Bandwith 104 Hz). The Biosemi active electrode system uses active online referencing through a Common Mode Sense electrode.

### Gambling task

**Figure 1:**
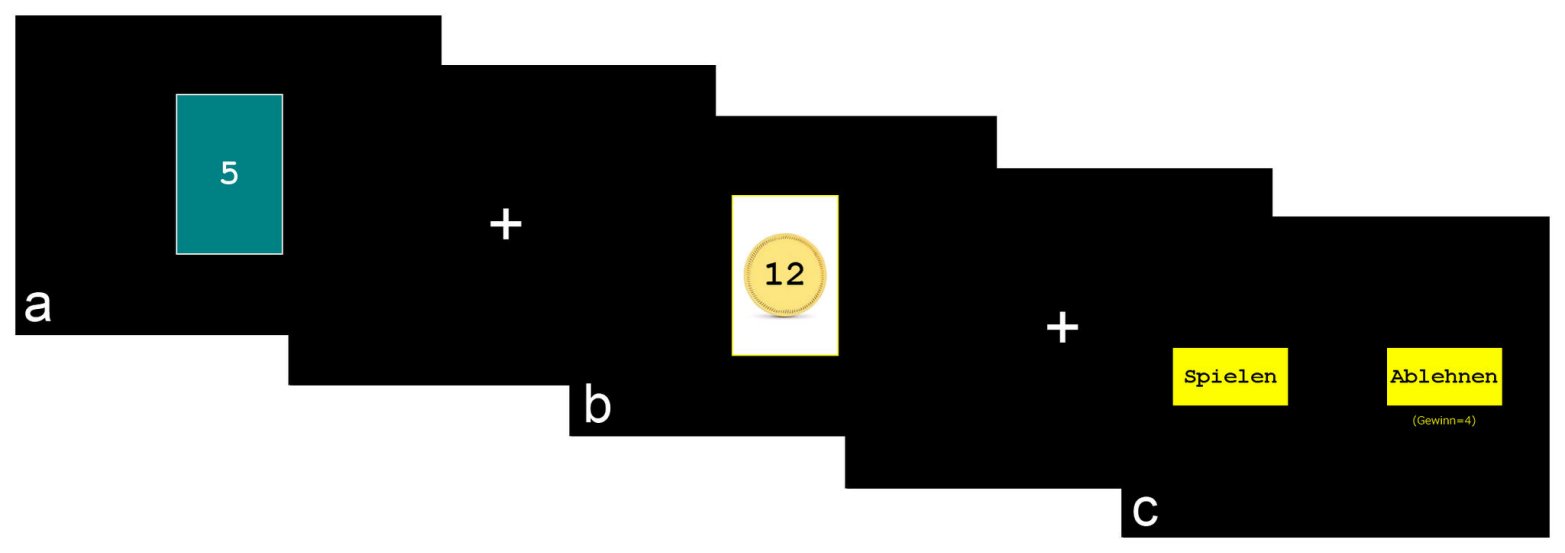
Experimental Task. On each trial, participants were first presented with a card indicating the chances of winning (a) and then with the potential win amount (b). Next, participants chose to accept the gamble (“spielen”), or reject it (“ablehnen”) and go for a small certain win (c).

The computerized experimental task was programmed using E-Prime (Version 2, Psychology Software Tools Inc, Pittsburgh, USA). On each trial, participants were presented with a gamble and decided whether to accept it or go for a small certain gain instead. Each gamble yielded the chance of an attractive win, but could also result in winning nothing. Gambles were presented to participants in the form of a card game, with the following rules: On each round, two cards are being drawn sequentially by the computer from a deck of 11 cards with the number 0-10 on them. If the second card is higher than the first, the participant wins; if the second card is lower than the first the participant loses and receives nothing. Thus, the first card represents the chances of winning (for example, a card of 6 indicates a 40% chance of winning). On each trial, participants were first presented with the first card and then saw the potential win amount (see Figure 1). Six different gambles were created by combining three different probabilities of winnings (30%, 40% or 50%) and two different potential win amounts (9 or 12 CHF). The small certain gain was identical in all trials (4 CHF). Participants were explicitly informed about the exclusive occurrence of these odds and win amounts before the task. Participants indicated their choice (‘play’ or ‘reject’) by pressing a corresponding key on a computer keyboard. In the majority of trials, the outcome of the gamble (i.e. the second card) was not presented to participants to limit the influence of the decision outcome on subsequent decision. To ensure that participants kept engaged in the task, the outcome was however displayed for 25% of the accepted gambles. Each gamble was presented to the participant 30 times, resulting in a total of 180 trials (in addition to 5 practice trials). At the end of the experiment, one trial was randomly chosen to determine the real bonus amount (depending on the participant’s choice and the outcome of the gamble). Since payment was dependent on a single choice, participants could not hedge risk across trials.

### EEG data processing

Offline pre-processing of EEG data was conducted using Brain Vision Analyzer software (Version 2; Brain Products Gmbh, Germany). A low-pass filter of 40Hz, a high-pass filter of 1 Hz and a Notch filter (50Hz) were applied. EEG data was downsampled to 256Hz, and channels with excessive noise (due to malfunctioning or bad signal during data collection) were replaced using topographic interpolation. Independent component analysis (ICA) was computed to correct for eye-movement artifacts, and EEG data was visually inspected for other artifacts. Five participants had to be excluded from further analysis due to extensive artifacts in their EEG recordings. Thus, statistical analysis was conducted on the data of 65 participants (average of 171 ± 22 seconds of artifact-free EEG data). Next, EEG data was re-referenced to common average and segmented into eyes-closed and eyes-open periods. Several authors [26,27] have recommended that sensory input should be kept as minimal as possible during resting-state recordings. Therefore only eyes-closed periods were analyzed. Each artifact-free eyes-closed period was further segmented into epochs of 2 seconds (512 data points).

The data were then exported into standardized low-resolution brain electromagnetic tomography software (sLORETA; Pascual-Marqui, 2002) for calculation of intracortical power spectra. First, fast Fourier transformation (using a square window) was applied to scalp EEG data to estimate absolute spectral power averaged over all epochs in the δ (1–3 Hz) and θ (4–7 Hz) frequency bands. We focused on the slow-wave oscillations (i.e. δ and θ) given that these frequencies have previously been linked to risk-taking behavior [9,25] (An explorative analysis of the α (8–12 Hz), β1 (13–18 Hz) and β2 (19–21 Hz) frequency bands can be found in File S1 (see Tables S1 and S2 in File S1). Intracerebral electrical sources generating the scalp-recorded activity in these frequency bands were estimated by computing intracranial current density (A/m^2^) as implemented in sLORETA. The sLORETA solution space is based on the Montreal Neurological Institute atlas and is restricted to 6239 voxels in cortical grey matter. Current densities for the δ and θ frequency bands were then averaged across the voxels of the right and left prefrontal cortex (Brodmann areas 8, 9, 10, 11, 44, 45, 46, 47), and normalized by dividing the averaged current density for each band by the averaged total current density (1-30Hz), separately for each site. Two measures for each frequency were calculated: a) resting-state activity in the PFC overall (left + right) and b) PFC asymmetry index (right-left). These measures were z-transformed and entered into statistical analysis (A visualization of PFC δ and θ activity is provided in Figure S1 in File S1).

### Statistical analysis

Statistical analysis was carried out in SPSS (Version 20, IBM Corporation). In a first step, we investigate the relationship between risk-taking behavior on the task and prefrontal activity during resting state. For each participant, the percentage of risky choices (i.e. accepting the gamble) was calculated. Pearson’s correlations between this measurement of risk-taking and a) relative power of slow-waves (i.e. δ and θ oscillations) in the overall PFC, and b) the asymmetry indexes for the δ- and θ-frequency bands were calculated. In a second step, we tested whether individual differences in BIS/BAS scores were related to heterogeneity in risk-taking behavior. Pearson’s correlations between the percentage of risky choice and participants scores on the BIS and BAS scales were calculated. In a third step, we assessed whether differences in personality were correlated with PFC activity during rest by calculating Pearson’s correlations between BIS/BAS scores and the EEG measures. All correlations were conducted two-tailed, and P-values were corrected for multiple comparisons using the Hochberg procedure [28].

In a final step, we assessed whether resting-state PFC activity and personality function as independent predictors, or whether they interact in determining risk-taking behavior. The aforementioned correlation analyses revealed that BIS scores and PFC asymmetry index for the θ-band were significantly correlated with risk taking on the task (see Results section for details). To investigate the relationship between these predictors, a multiple linear regression was calculated to predict risk taking on the task (i.e. percentage of risky choices) from BIS scores, PFC asymmetry index for the θ-band and the interaction of these two variables (multiplication of the two z-transformed measures).

## Results

### Risk-taking behavior

**Figure 2:**
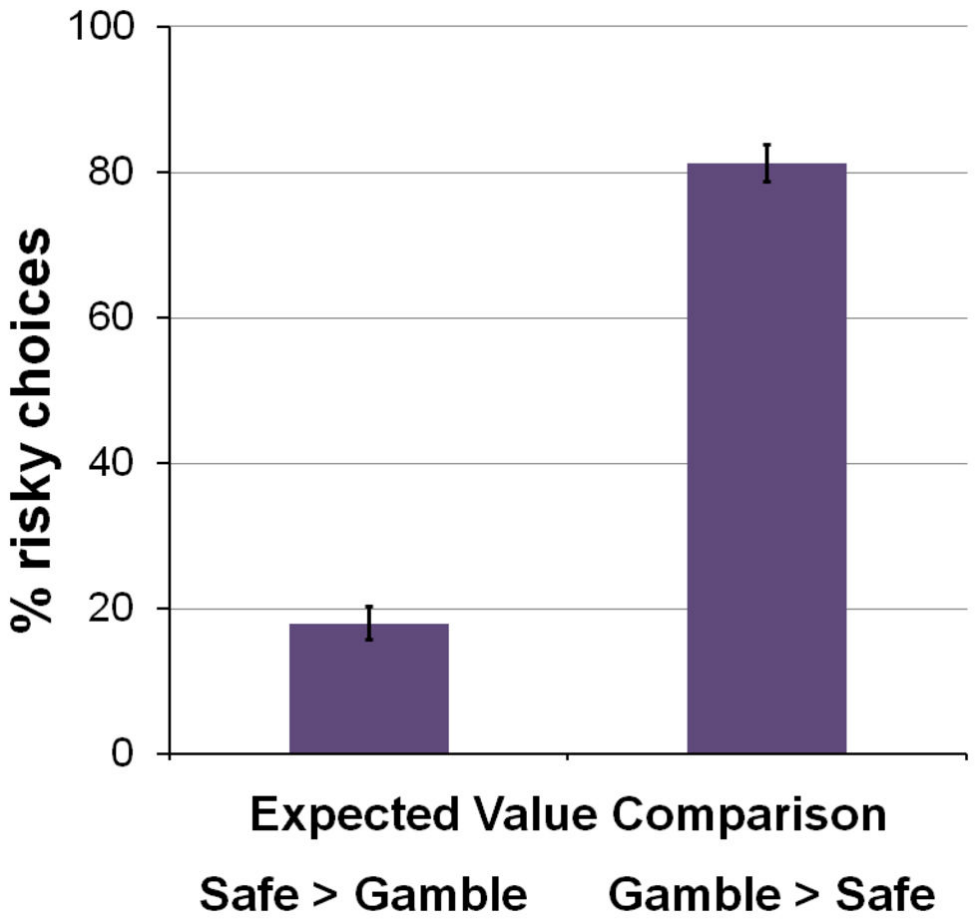
Choice Behavior. When the gamble was lower in expected value than the certain win, participants chose the risky option (i.e. accepted the gamble) far less often than when the expected value of the gamble was higher than the certain win (M_Gamble<Safe_=18%, M_Gamble>Safe_=82%, T=-19.65, P<.00001).

On average, participants chose to accept the gamble in 50% of trials (SD = 14%). Participants demonstrated a high sensitivity to the gambles’ expected value as compared to the certain win in their decisions (see Figure 2 and Table 1). Most importantly, and as expected, considerable individual differences in risk-taking behavior were observed, with the proportion of risky choices ranging from 10% to 82% across participants.

**Table 1:** Average percent of risky choices on each trial type.

**Gamble**			**Accept Percentage**	
*Chances of winning*	*Win Amount*	*Expected Value*	*Mean*	*SEM*
30%	9	2.7	6%	2%
30%	12	3.6	27%	4%
40%	9	3.6	20%	3%
40%	12	4.8	73%	4%
50%	9	4.5	78%	3%
50%	12	6	92%	2%

### Relationship between PFC resting-state activity and risk-taking behavior

We first examined whether heterogeneity in risk-taking behavior can be associated with individual differences in resting-state PFC activity. The PFC asymmetry index for the θ-band was positively correlated with the proportion of risky choice in the task (R=.34, P=.02). Individuals with higher θ-band power in the right compared to the left PFC took more risks on the task. While PFC asymmetry in the δ-band also tended to correlate with the proportion of risky choices, this relationship was not statistically significant after correction for multiple comparisons (R=.26, P=.13, unadjusted P=.04). Power of slow-wave oscillations in the bilateral PFC overall was not significantly correlated with the proportion of risky choice (δ: R=.17, P=.32; θ: R=.02, P=.85). A follow-up analysis of slow-wave power in the right and left PFC separately showed no significant correlations with proportion of risky choice either (see Analysis S2 in the File S1 for details). Thus, risk-taking behavior was specifically linked to asymmetry in resting-state activity in the right versus the left PFC.

### Relationship between BIS/BAS scores and risk-taking behavior

**Figure 3:**
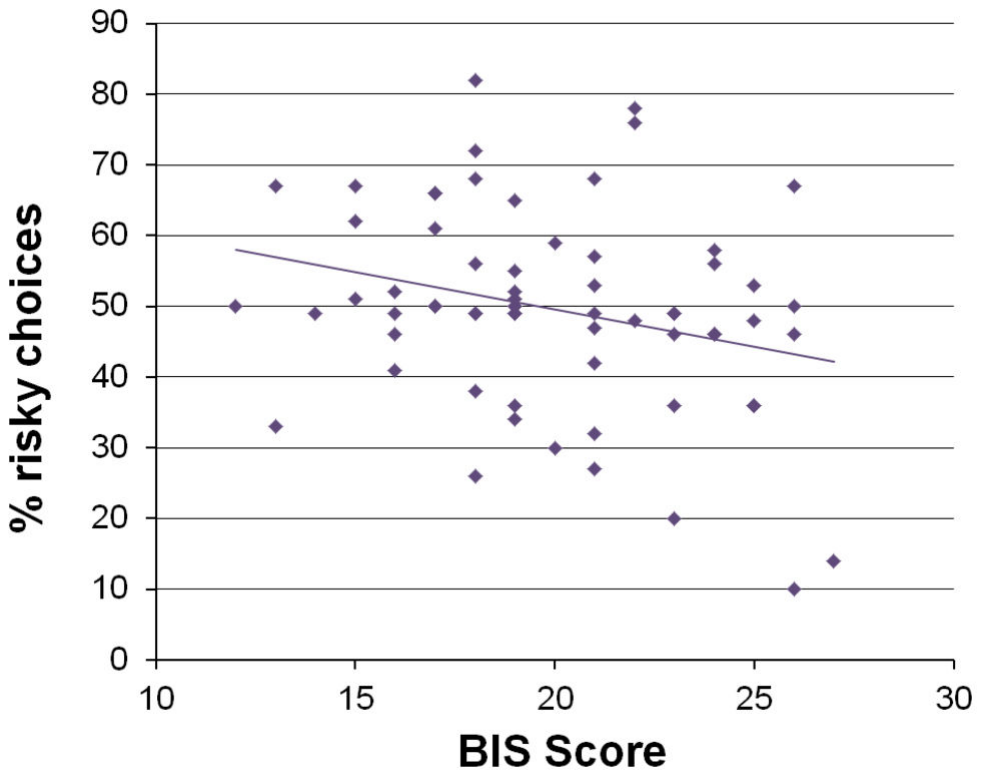
Trait sensitivity to punishment and risk-taking behavior. Scores on the BIS scale were negatively correlated with the level of risk-taking on the task (R=-.27, P<.05).

The observed heterogeneity in risk-taking behavior could also be linked to individual differences in personality characteristics. The proportion of risky choices was negatively correlated with scores on the BIS scale across participants (R=-.27, P=.05). That is to say, individuals with higher sensitivity to punishments (i.e. high BIS scores) made more risk-averse decisions on the task than individuals with low BIS scores (see Figure 3). No significant correlation between scores on the BAS scale and risk-taking was found (R=-.14, P=.23). A follow-up analysis further found that scores on the BIS and BAS scale were not significantly correlated with each other (R=.13, P=.30).

### Relationship between BIS/BAS scores and EEG resting state

In a next step, we tested whether BIS/BAS scores and PFC activity during rest were directly related to each other. Neither the δ- and θ-band asymmetry indices nor δ- and θ-band power in the bilateral PFC were significantly correlated with BIS scores (all Ps>.7, largest R=-.17). Likewise, no significant correlations between the EEG resting-state measures and BAS scores were found (all Ps>.18, largest R =.24). In conclusion, these results do not show a relationship between the personality measure and PFC resting-state activity.

### Predicting risk-taking behavior from resting-state PFC activity and personality

**Figure 4:**
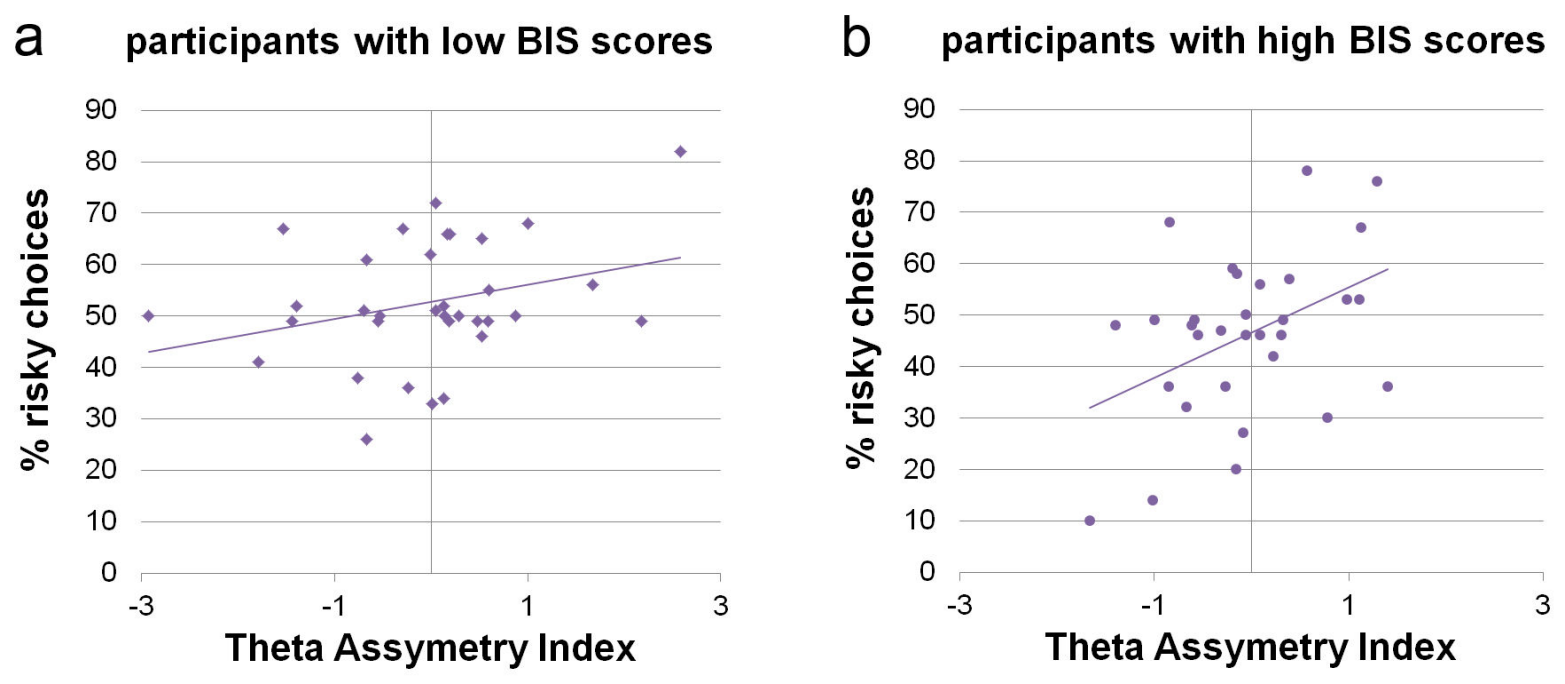
Asymmetry in prefrontal resting-state activity and risk-taking behavior. Right-left asymmetry in intracranial theta band-power during rest was correlated with the level of risk-taking on the task. This relationship was stronger in participants with high BIS scores (b) than in participants with low BIS scores (a). For the purpose of this illustration only, a medium split has been used to divide the sample into low and high BIS groups.

In summary, both asymmetry in PFC resting-state activity and punishment sensitivity as assessed by the BIS scale predicted individual differences in risk-taking. In a final step, we assessed whether BIS scores and PFC asymmetry index for the θ-band interacted to determine risk-taking behavior. A multiple regression analysis confirmed the degree of asymmetry of θ-band power in the right versus left PFC and BIS scores as significant predictors of risky choice behavior, and additionally revealed a marginally significant interaction between BIS scores and the PFC asymmetry index (see Table 2 and Figure 4). Moderation analysis [29] assessing the conditional effect of prefrontal resting-state asymmetry (θ-band power) resulted in a beta value of .25, .43 and .62 for low (mean - 1SD), average and high (mean + 1SD) BIS scores respectively. Thus, the relationship between PFC resting-state activity and risk-taking behavior was modulated by BIS scores (i.e. trait sensitivity to punishment). In other words, left-right asymmetry in PFC activity at rest was found to have a stronger predictive power in participants with high BIS scores compared to low BIS scores. Together, asymmetry in PFC θ-band power, BIS scores and their interaction explained 25% of the variance in risk-taking behavior. Cross-validation analysis confirmed these results (for details see Analysis S3 in File S1).

**Table 2:** Results of regression analysis.

*Dependent variable =* **Proportion of risky choice**			
*Predictors*	*Results*		
	*Beta (Std.)*	*T-Value*	*P-Value*
**θ Asymmetry Index**	**.42**	**3.63**	**.001**
**BIS scores**	**-.23**	**-2.06**	**.04**
**θ Asymmetry Index x BIS score**	**.19**	**1.78**	**.08**

Overall regression model: R=.50, R^2^=.25, DF=3, N=65, F=6.68, P<.001

## Discussion

The current study examined the relationships between individual differences in risk-taking behavior, personality traits and basic physiological properties of the brain. We measured participants’ risk-taking behavior with an economic decision-making task, and related it to their scores on the BIS/BAS scale and resting-state oscillatory activity in the PFC. The study revealed three main findings: First, individual differences in risky choice behavior could be predicted by hemispherical balance in PFC resting-state activity. Higher theta-band power in the right compared to the left PFC was associated with increased risk taking. Second, our data confirmed that heterogeneity in risk-taking behavior is linked to differences in personality. Scores on the BIS scale were negatively correlated with the level of risk taking across participants. Third, these two different predictors (BIS scores and theta-band asymmetry in the PFC at rest) were not directly linked, but interacted in predicting risk-taking behavior. The relationship between PFC resting-state asymmetry and risky choice behavior was stronger in participants with higher scores on the BIS scale.

The first aim of the current study was to test whether risk taking can be predicted by resting-state activity in the prefrontal cortex. More specifically, we aimed to clarify whether risky choice is associated with activity in the bilateral prefrontal cortex or with the hemispherical balance in prefrontal activity at rest. We found that right-left asymmetry in PFC slow-wave activity predicted risk taking: Participants with stronger theta-band power in the right compared to the left PFC took more risks on the subsequent decision-making task. Our finding aligns with Gianotti et al. [9], who reported a correlation between asymmetry in PFC slow-wave power at rest and risk-taking on the Devils Task [11]. Our result indicates that this relationship generalizes to different measures of risk-taking behavior (see also [30,31] on why this is important): The Devil’s task assesses risk-taking in a sequential, dynamic choice situation, while we used a non-sequential, static decision-making task [30]. No significant relationships between the level of risk taking on our task and overall resting-state activity in the bilateral PFC, or in the right and left PFC alone, were found. Thus, individual differences in risky choice behavior were specifically linked to the *hemispheric balance* in PFC theta-band activity at rest.

The relationship between EEG frequency bands and cerebral activity is complex, and is likely to vary between different regions of the brain [32,33]. In the case of the prefrontal cortex, several previous studies have found an inverse correlation between theta-band activity during resting-state recordings and glucose metabolism [32] and blood-oxygen-level-dependent signal [33-35] (but see also [36]). These studies thus suggest that high prefrontal theta-power at rest indicates a low level of neural activity. In the current study, increased risk taking was associated with higher theta band power (corresponding to lower activity) in the right versus left PFC. Theories of emotion and motivation attribute specific and opposed functions to the two hemispheres of the prefrontal cortices: the right PFC is thought to drive withdrawal behavior, while the left PFC promotes approach behavior [37-40]. One explanations of our results could be that tonic left-lateralized PFC activity leads to increased attention to potential positive outcomes and reduced attention to potential negative outcomes in a decision situation (see also [41,42]). Future research might test this interpretation by assessing whether the relationship with risk-taking behavior replicates when PFC resting-state activity is assessed by vascular or metabolic measures of brain activity.

Individual differences in risk taking were also explained by heterogeneity in personality. Participants with higher scores on the BIS scale made fewer risky choices on the task than participants with low BIS scores. This result was expected, given that the BIS scale measures trait sensitivity to potential punishment/loss, and accepting the gamble in our task meant to lose out on the small certain win and risk walking away with nothing. The current result aligns with prior data linking BIS scores to decision-making performance on the IGT [15-18] and sensitivity to the odds of winning on the Roulette Betting Task [19]. In addition, we expected to find a positive association between scores on the BAS and the level of risk taking, based on two previous studies reporting a correlation between performance on the IGT and BAS scores [15,17]. However, no such relationship was observed in our data (see also [5]). This inconsistency in extant findings suggest that the relative importance of sensitivity to reinforcement (i.e the BIS) and sensitivity to punishment (i.e. the BAS) might depend upon the nature of the task used to assess risk-taking behavior: The BAS may be more strongly associated with choice behavior in dynamic tasks such as the IGT, while risk-taking on static, economic task like ours may be more strongly influence by the BIS. Future research should test this hypothesis directly.

The current study further aimed to investigate how these personality and brain-physiological predictors of risk-taking behavior relate to each other. More specifically, we tested whether PFC resting-state activity and scores on the BIS/BAS scale were directly correlated, or whether they interacted to predict risk-taking behavior. No significant correlations between BIS/BAS scores and bilateral PFC activity or asymmetry in PFC activity at rest emerged in our sample. This result contrasts with previous studies reporting a correlation between resting-state alpha band activity in frontal electrodes and scores on the BAS [21-24] and BIS [20,21] scales. One potential explanation for this discrepancy with our findings is that resting-state alpha and theta activity reflect different functional properties of the prefrontal cortex. Differences in the methodology between the current and prior studies might also play a role. In particular, we used source modeling to calculate intracortical electrical activity within the PFC, while the aforementioned studies have analyzed activity in frontal electrodes to estimate resting-state activity of the underlying cortex (however, see [20] for an exception). We did observe a marginally significant interaction effect between resting-state prefrontal activity and sensitivity to punishment in predicting risk-taking behavior, in addition to independent effects of each predictor. The link between resting-state asymmetry of the PFC and risk-taking behavior was stronger for participants with a higher scores on the BIS scale. In close parallel, Massar and colleagues [25] recently reported that resting-state activity in fronto-central electrodes predicted risk-taking following a high win on a laboratory gambling task in participants with high, but not low, BIS scores. The current study replicates this recent finding and demonstrates that the prefrontal cortex is the generator of the resting-state activity predicting risk-taking behavior. What might be the mechanisms underlying such an interaction of sensitivity to punishments measured by the BIS and resting-state prefrontal activity in predicting risk taking? The BIS was defined as a neurological motivation system comprising structures of the limbic system [43], while the prefrontal cortex is known to be involved in behavioral control and implicated in the mediation of motivational systems [44-47]. It is thus conceivable that the observed interaction effect might reflect the interplay between a subcortical motivation system and a prefrontal control system. It would be interesting for future research to test whether basic connectivity between limbic subcortical structures and prefrontal cortex can predict risk-taking, for example by using resting-state functional magnetic resonance imaging.

Together, BIS scores and asymmetry in resting-state PFC activity explained 25% of the between-subject variance in risk-taking behavior on our task. A significant portion of the variance in risky choice thus remained unaccounted for. Future research should aim to identify additional sources of the heterogeneity in risk-taking behavior. For example, a limitation of EEG is that it primarily measures activity of brain structures on the cortical surface. Differences in the baseline activity of subcortical structures are likely to also influence reward-guided behavior and risk taking. In the current study, we focused purely on local resting-state activity. Future research might assess whether heterogeneity in structural and functional connectivity in the large brain network underling choice behavior can help explain further variance in risk taking.

In conclusion, the current study demonstrates that individual differences in risk-taking behavior are linked to a person’s sensitivity to punishment and hemispherical balance in resting-state activity of the prefrontal cortex. Furthermore, sensitivity to punishment and prefrontal physiology were found to interplay in predicting risk-taking behavior.

## Supporting Information

File S1 **Supporting files**.
Analysis S1, Resting-state power in the alpha, beta1 and beta 2 frequency bands. Table S1, Correlations between risk taking and EEG resting-state measures. Table S2, Correlations between risk taking and scores on the BIS and BAS scales. Analysis S2, Unilateral theta and delta band power at rest. Analysis S3, Cross-validation of results of regression analysis. Figure S1, Visualization of resting-state theta and delta band activity in the prefrontal cortex.
(DOCX)
