# Supplementary material for: Predicting Risk-Taking Behavior from Prefrontal Resting-State Activity and Personality
Source: PLoS One. 2013 Oct 7;8(10):e76861. doi: 10.1371/journal.pone.0076861 (PMC3792091; doi:10.1371/journal.pone.0076861)
Supplement: File S1 — Supporting files. Analysis S1, Resting-state power in the alpha, beta1 and beta 2 frequency bands. Table S1, Correlations between risk taking and EEG resting-state measures. Table S2, Correlations between risk taking and scores on the BIS and BAS scales. Analysis S2, Unilateral theta and delta band power at rest. Analysis S3, Cross-validation of results of regression analysis. Figure S1, Visualization of resting-state theta and delta band activity in the prefrontal cortex. (DOCX) [file pone.0076861.s001.docx]

## File S1: Supporting Information

## Manuscript: Predicting risk-taking behavior from prefrontal resting-state activity and personality

**Authors:** Bettina Studer, Andreas Pedroni, Jörg Rieskamp

Supplementary analyses

Analysis S1: Resting-state power in the alpha, beta1 and beta 2 frequency bands

Previous research has found that risk-taking behavior is specifically associated with prefrontal resting-state power in the delta and theta frequency bands, and thus we focused on these slow-wave oscillations. To supplement the findings reported in the main manuscript, we also conducted explorative analyses of the relationships between resting-state PFC power in the α (8–12 Hz), β1 (13–18 Hz) and β2 (19–21 Hz) frequency bands and i) risk taking behavior and ii) scores on the BIS/BAS scale. Intracranial current density were calculated for each frequency band, averaged across the voxels of the right and left prefrontal cortex, and normalized. Next, two measures were calculated for each frequency: a) resting-state activity in the PFC overall (left + right) and b) PFC asymmetry index (right - left). These measures were z-transformed and entered into statistical analysis. Pearson’s correlations between these indexes of PFC resting-state activity and i) percentage of risky choices and ii) BIS and BAS scores were calculated. All statistical tests were conducted two-tailed.

For the α, β1 or β2 frequency, risk taking on the task was not significantly correlated with bilateral PFC power or the PFC asymmetry index for any of these frequency bands (see Table S1 for details).

**Table S1: Correlations between risk taking and EEG resting-state measures**

|  | **Alpha** | | **Beta 1** | | **Beta 2** | |
| --- | --- | --- | --- | --- | --- | --- |
|  | r | P | r | P | r | P |
| **Asymmetry Index** | .10 | .43 | .07 | .55 | .12 | .33 |
| **Bilateral PFC power** | -.20 | .11 | -.21 | .10 | -.14 | .25 |

r = Pearson’s correlation coefficient. Uncorrected p-values are reported.

Similarly, no significant relationships between scores on the BIS/BAS and bilateral PFC activity or PFC asymmetry indexes for the α, β1 or β2 frequency were found (see Table S2 for details).

**Table S2: Correlations between risk taking and scores on the BIS and BAS scales**

|  | ***BIS scores*** | | | | | | ***BAS scores*** | | | | | |
| --- | --- | --- | --- | --- | --- | --- | --- | --- | --- | --- | --- | --- |
|  | **Alpha** | | **Beta 1** | | **Beta 2** | | **Alpha** | | **Beta 1** | | **Beta 2** | |
|  | r | P | r | P | r | P | r | P | r | P | r | P |
| **Asymmetry Index** | .02 | .85 | .07 | .55 | -.01 | .94 | .15 | .24 | .01 | .93 | .19 | .23 |
| **Bilateral PFC power** | .19 | .13 | .17 | .17 | .16 | .19 | .17 | .18 | .15 | .23 | .02 | .89 |

r = Pearson’s correlation coefficient. Uncorrected p-values are reported.

In summary, we found no statically significant correlations between resting-state EEG measures for the α, β1 or β2 frequency bands and i) risk taking or ii) scores on the BIS/BAS scale in our sample.

Analysis S2: Unilateral theta and delta band power at rest

As reported in the main manuscript, individual differences in risk taking were significantly associated with hemispherical asymmetry in resting-state PFC activity (θ band), but not with the overall, bilateral PFC activity level. To complement these results, we conducted Pearson’s correlations between risk taking on the task and θ- and δ-power in the right and left PFC alone. P-values were corrected for multiple comparisons using the Hochberg procedure. No significant differences between the proportion of risky choice in the task and slow-wave power in the right or left PFC alone were found (left PFC: δ: R=.14, P=.80; θ: R=-.07, P=.61, right PFC: δ: R=.19, P=.53; θ: R=.12, P=.67).

Analysis S3: Cross-validation of results of regression analysis

In order to examine the reliability of the regression results reported in the main manuscript, a repeated cross-validation analysis was conducted. Cross-validation involves estimating regression weights in one subset of data (referred to as ‘training set’) and then testing them on a second subset of data (referred to as ‘validation set’). We repeated this analysis ten times. Each time, we split the overall sample into two equally-sized subgroups, and performed the multiple regression analysis, with z-transformed BIS scores, θ-band asymmetry indexes and their interaction term as predictors and percentage of risky choices as a dependent variable, in the training set only. The obtained regression weights were then used to predict the level of risk taking for each participant in the validation set. Next, Pearson’s correlation between the predicted values and the observed risk taking was conducted.

On average, a correlation of medium size was found between predicted and observed levels of risk-taking in the valuation set (average Pearson’s r = .32, SD = .20, n = 32-33). On average, the observed percentage of risky choices fell within the 95% Confidence Interval for the predicted value for 97% of subjects in the validation set. In summary, while there was some variability in the results obtained in different rounds, overall the analysis confirmed the initial regression results reported in the main manuscript.

Supplementary figures


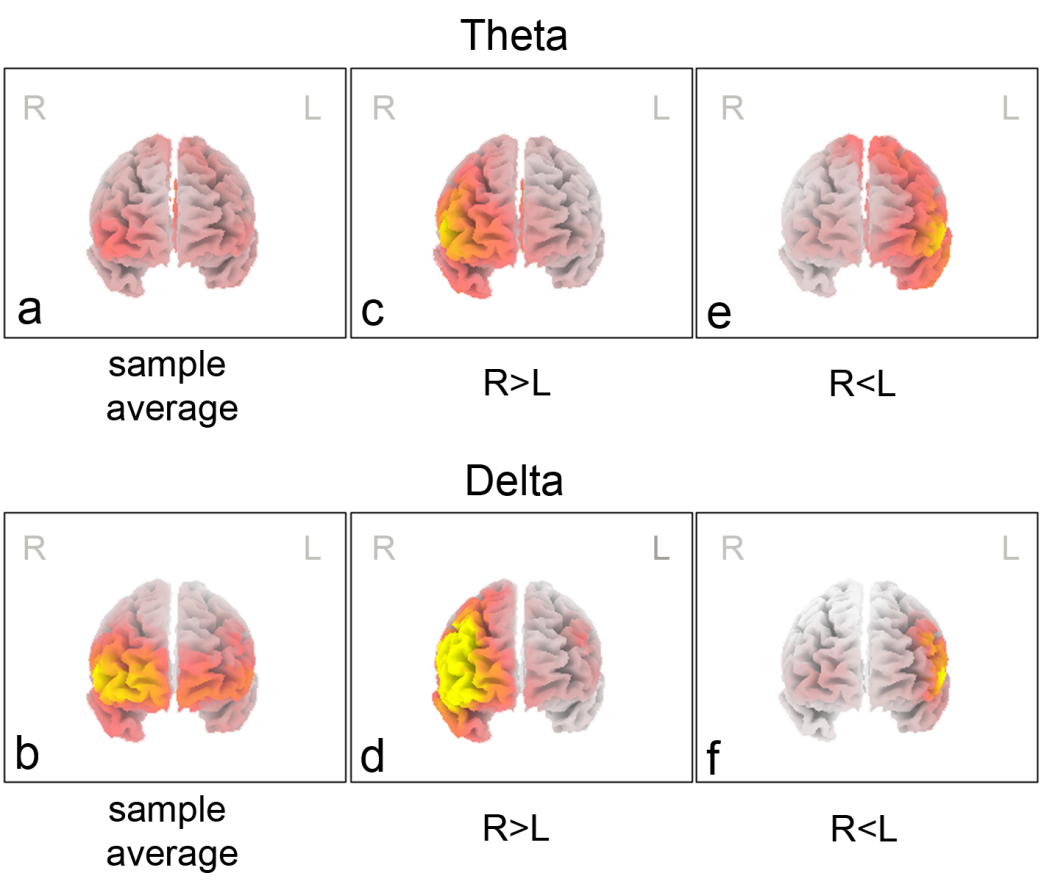


**Figure S1 – Visualization of resting-state theta and delta band activity in the prefrontal cortex (PFC).** Current densities in the PFC during rest are shown in the graphics. Figures a and b show the resting-state activity in the full sample (n=65). For the remaining figures, the sample was divided into three equally sized subgroups according to their asymmetry index. Figures c and d show the activity in a subsample (n=21) with positive asymmetry indexes, that is to say, with higher theta and delta power in the right compared to the left PFC. Figured e and f show the activity in a subsample (n=22) with negative asymmetry indexes, that is to say, with higher theta and delta power in the left compared to the right PFC.
